# Comparative Late Effects of Hemostatic Biomaterials on Wound Healing at 14 and 30 Days: An In Vivo Animal Study

**DOI:** 10.3390/jfb17040183

**Published:** 2026-04-09

**Authors:** Polina Shabes, Julian-Dario Rembe, Arzu Mammadova, Katharina Henrika Beckamp, Markus Udo Wagenhäuser, Wiebke Ibing, Hubert Schelzig, Waseem Garabet

**Affiliations:** Department of Vascular and Endovascular Surgery, University Hospital of Duesseldorf, Heinrich-Heine University (HHU), 40225 Düsseldorf, Germanyjulian-dario.rembe@med.uni-duesseldorf.de (J.-D.R.);

**Keywords:** hemostatic biomaterials, wound healing, growth factors, oxidized cellulose, gelatin

## Abstract

Hemostatic biomaterial agents are widely used during surgery and trauma care to control bleeding, yet their effects on wound healing remain incompletely understood. This study evaluated the impact of oxidized non-regenerated cellulose (ONRC), oxidized regenerated cellulose (ORC), and a gelatin-based hemostat (GELA) on wound healing at 14 and 30 days in a mouse model. Full-thickness wounds were created in C57BL/6J mice (*n* = 192) and compared to sham controls. Tissue samples were analyzed histologically, supported by immunohistochemistry for Ki-67 and α-SMA and qPCR for VEGF, TGF-β, and FGF-2. Histology demonstrated preserved tissue architecture across groups with progressive resorption of cellulose-based materials, whereas GELA showed localized fibrous structures and enhanced extracellular matrix formation. At day 14, no significant differences were observed in proliferation, contraction, VEGF, or FGF-2 expression; however, TGF-β was significantly reduced in the ORC group. By day 30, GELA significantly increased epidermal proliferation, while contraction markers were elevated in both GELA and ORC. VEGF expression was reduced in GELA and ORC, whereas ONRC showed increased TGF-β expression. FGF-2 remained unchanged across groups. All investigated hemostatic materials were well tolerated during the early postoperative phase (up to day 14), indicating short-term biocompatibility within the scope of this model. In contrast, material-specific differences in cellular activity and growth factor expression became apparent during the later remodeling phase (day 30). These findings suggest differential effects on cellular and molecular aspects of tissue remodeling; however, no conclusions can be drawn regarding overall healing quality or clinical safety, as no quantitative macroscopic or functional outcome measures were assessed.

## 1. Introduction

Hemostatic biomaterials such as oxidized regenerated cellulose (ORC), oxidized non-regenerated cellulose (ONRC), gelatin-based matrices (GELAs), and chitosan- or collagen-based dressings are commonly used to achieve hemostasis during surgical procedures [1].

Due to their heterogenous efficacy and safety, hemostats have been compared in several previous studies, primarily regarding hemostatic properties, but also in terms of wound healing [2,3,4,5,6]. As hemostatic biomaterials may exert both positive [3,5,6] and negative effects on wound healing [4,7,8], especially if left in situ, great care must be taken regarding their appropriate use to avoid post-surgical wound-healing disorders (WHDs).

WHDs can negatively affect a patient’s outcome and prognosis after surgical treatment. As well as prolonged hospitalization and reduced quality of life, WHDs can escalate to serious complications such as sepsis or erosive bleeding resulting in hemorrhagic shock. Excessive intra- and postoperative bleeding with subsequent hematoma formation and potential complications such as secondary infection and erosive bleeding, pain, or tissue damage due to local pressure can already result in WHDs or the need for re-operation. To control local bleeding in surgical procedures and trauma management, hemostatic agents are frequently employed and left in situ at the site of wounding [1].

The long-term effects of topical absorbable hemostatic agents on wound healing remain insufficiently explored, however, and are the subject of ongoing debate. Although these materials are routinely employed for intraoperative bleeding control, their role in tissue regeneration, fibrosis formation, and chronic inflammatory responses is not yet clearly defined. While the immediate need to prevent blood loss typically outweighs concerns about secondary complications such as WHDs, current knowledge on the subsequent impact of hemostats during the healing phase remains limited and often contradictory.

The course of wound healing can be divided into coordinated and overlapping phases: hemostasis, inflammatory phase, proliferative phase including formation of granulation tissue and angiogenesis, re-epithelialization, and remodeling phase [9,10]. The wound-healing process is controlled by various immunomarkers such as growth factors and cytokines. Growth factors induce extracellular matrix (ECM) formation by stimulating regenerative cell types such as fibroblasts and endothelial cells and regulating the production of matrix-degrading proteases and their inhibitors [11]. Relevant growth factors include vascular endothelial growth factor (VEGF), which plays an important role in angiogenesis, inducing proliferation and chemotaxis in endothelial cells [12], and fibroblast growth factor 2 (FGF-2), which also stimulates endothelial cell proliferation, contributing to angiogenesis, and targets vascular smooth muscle cells and fibroblasts to induce collagen synthesis, wound contraction, epithelialization, and fibronectin and proteoglycan synthesis [13]. Further key factors and markers include transforming growth factor beta (TGF-ß), which activates angiogenesis, proliferation, and collagen synthesis in fibroblasts [14,15]; alpha smooth muscle actin (α-SMA), which plays a central role in wound contraction and ECM remodeling exerted by myofibroblasts and induces fibrogenic changes [16,17,18]; and Ki-67, a nuclear protein expressed during all active phases of the cell cycle (G_1_, S, G_2_, and M) but not in resting cells (G_0_), making it an established cellular marker of cell proliferation (primarily used in the context of cancer diagnosis and therapy) [19,20,21,22].

While the situationally appropriate use of hemostats can prevent the development of post-surgical WHDs by aiding the hemostatic phase of healing, their effect on the cellular processes of wound healing has only been investigated to a limited extent, demonstrating that different hemostatic agents can influence the abovementioned processes both positively and negatively [4,23]. ORC (within the range of 16–24% 6-carboxyl acid content), for example, shows excellent hemostatic effects, degrades to non-toxic material at the site of use within several weeks, and shows promising pro-healing properties [24]. GELAs have also exhibited promising in vitro results in wound healing and clinical hemostatic properties, partly superior to ORC or ONRC, and could therefore be an innovative alternative hemostatic material [4,8,25,26].

A previous study by our research group investigated the early effects of hemostatic agents in vivo [27]; however, in vivo investigations of the late effects of hemostatic biomaterials in a side-by-side comparison are so far lacking. This study helps to close this gap by investigating the cellular and biomolecular effects of three hemostatic agents (ONRC, ORC, and GELA) on wound healing in a mouse model. The hemostatic agents were compared to a control group without hemostat treatment for up to 30 days. Such a comparative approach may help to better understand material-specific differences in wound healing and support more informed selection of hemostatic agents in clinical practice.

## 2. Methods

### 2.1. Investigated Hemostatic Biomaterials

Three hemostatic agents were investigated (Table 1): two cellulose-based dressings (ONRC and ORC) and a porcine GELA. Cellulose is a plant-derived, biodegradable material, generally part of the primary cell wall in green plants. It is a homopolysaccharide of glucopyranose polymerized through β-glycosidic bonds. When cellulose is regenerated, organized fibers are formed, while in non-regenerated cellulose fibers stay unorganized prior to oxidation [6]. Gelatine is a hydrocolloid made from partial acid hydrolysis of porcine-derived collagen that is whipped into foam and then dried [2]. According to the manufacturers, all three dressings are absorbable: ONRC and ORC are absorbed after 14 days; GELA should be absorbed within 30 days.

The selected hemostatic biomaterials represent clinically established products with distinct structural and compositional properties. ONRC consists of non-regenerated oxidized cellulose with a more heterogeneous and less organized fiber architecture, whereas ORC is composed of regenerated oxidized cellulose characterized by a more structured and uniform fiber arrangement. GELA is a porcine-derived gelatin-based matrix obtained through partial hydrolysis of collagen. These materials were chosen to represent different classes of absorbable hemostats with varying physicochemical properties, degradation kinetics, and potential interactions with surrounding tissue. This variety allows for a comparative assessment of how material composition and structure may influence cellular responses and tissue remodeling processes in a clinically relevant setting.

The selected time points of 14 and 30 days post-surgery were chosen to represent clinically meaningful stages of postoperative wound healing. Day 14 corresponds to the transition from the proliferative phase into early remodeling, at which point most surgical wounds are macroscopically closed. Day 30 represents a critical early remodeling window, during which it becomes apparent whether wound healing proceeds toward physiological maturation or shifts toward a pro-fibrotic or dysregulated remodeling trajectory.

For intraoperative use, 1 × 1 cm pieces of hemostatic agent were used per mouse.

### 2.2. Experimental Animal Model

The selection of the animal model was based equally on clinical relevance and practicality to allow for the highest possible translation of the laboratory experiments to the clinical setting. Therefore, a small animal model in the mouse was used in this experimental setup. To avoid cycle-related physiological variations, only fully grown male animals (*Mus musculus*, C57BL/6J, 8–16 weeks old) weighing 24–30 g were included. The study was reviewed and approved by the competent authority under the reference number [81-02.04.2022.A290], in accordance with the relevant regulations for animal experiments. All experiments were conducted following established guidelines and ethical standards for the care and use of laboratory animals. The study is reported in accordance with the ARRIVE guidelines (https://arriveguidelines.org).

The animals were obtained from Janvier Labs (Le Genest-Saint-Isle, France) and the local Central Institute for Animal Research and Scientific Animal Welfare (“Zentrale Einrichtung für Tierforschung und wissenschaftliche Tierschutzaufgaben”; ZETT; Düsseldorf, Germany). Animals with visible signs of illness or injury prior to surgery were excluded. No retrospective exclusions were made unless predefined criteria were met (e.g., unrelated unexpected health decline). Variations in sample size were due to layer-specific technical limitations.

### 2.3. Experimental Design and Surgical Procedure

Four groups of animals were investigated, including three intervention groups (one for each hemostatic agent to be tested) and a sham group as a comparison:

• **Group I: Sham** (control group);
  (animals undergoing surgery without implantation of *a* hemostatic agent);
• **Group II: GELA** (porcine gelatine hemostat);
• **Group III: ONRC** (oxidized non-regenerated cellulose);
• **Group IV: ORC** (oxidized regenerated cellulose).

Due to the different processing of tissues and samples, one group per test (*n* = 12) was required for each of the planned analyses. Two time points (14 and 30 days) for harvesting samples were evaluated for each group.

After the animals had settled in for at least one week, surgery was performed as follows. For the surgical procedure, animals were weighed and examined before receiving weight-adjusted anesthesia (ketamine/xylazine). Surgical preparation included shaving, disinfection, and sterile draping of the surgical field. During surgery, the animals were positioned on a heating table (37 °C), and a 2 cm skin incision was made in the midline of the back, extending through the skin and subcutaneous tissue to expose the underlying muscle fascia. A hemostatic agent (1 cm^2^) was applied subcutaneously onto the muscle fascia and left in situ. In the Sham group, the procedure was performed identically without application of a hemostatic agent. The wound was closed using Prolene 3-0 sutures, ensuring primary wound healing. This experimental model represents a closed incisional wound-healing model with primary intention, combined with subfascial implantation of a hemostatic biomaterial. Accordingly, the model can be defined as a “primary intention incisional healing with subfascial implant-induced tissue response.” In contrast to excisional or secondary intention wound models, the epidermis is primarily closed by sutures, and wound healing occurs predominantly within the dermal and subcutaneous compartments beneath an intact epithelial layer. Therefore, this model does not assess open wound closure dynamics, but rather the quality of tissue remodeling, cellular responses, and biomaterial–tissue interactions in a surgically closed environment.

On the first two days post-surgery, animals received standardized oral analgesia (1.33 mg/mL metamizole). Postoperative care included daily monitoring of animals and wounds during the first seven days, with behavior, general condition, and wound appearance assessed using a standardized scoring sheet provided by the animal facility. Thereafter, animals were monitored weekly until the respective experimental endpoint, and the experiment was discontinued if predefined criteria were met. Humane termination criteria (Table 2) were defined a priori and included close monitoring for signs of pain or distress. Animals were humanely euthanized in cases of body weight loss ≥20%, respiratory distress, paralysis, tail necrosis, severe bleeding, extensive wound infection, self-isolation, pain, or abnormal breathing sounds. Overall, the subcutaneous surgical procedure was classified as low stress, and anesthesia was administered prior to all invasive interventions.

Postoperative wound management was strictly standardized across all experimental groups, including anesthesia, analgesia, housing conditions, monitoring intervals, and predefined humane endpoints. Postoperative care was identical for all animals, irrespective of group allocation or time point, and no additional wound treatments were applied.

Wounds were photographed, and tissue samples were collected on postoperative days 14 and 30. At the experimental endpoints, animals were pre-medicated with metamizole and anesthetized with isoflurane prior to cardiac blood collection (maximum 1 mL). Euthanasia was performed by cervical dislocation, and wound tissue samples (2 × 2 cm, including cutis, subcutis, and connective tissue) were harvested for subsequent analyses.

### 2.4. Histologic and Immunohistochemistry Investigations

After fixation in 4% paraformaldehyde, tissue samples were embedded in kerosene and cut to a thickness of 4.5 µm. The sections were subsequently stained using hematoxylin-eosin staining (HE, MORPHISTO GmbH, Offenbach, Germany) and Movat Pentachrome staining (MP, MORPHISTO GmbH, Offenbach, Germany) and examined histologically according to standard protocols.

For immunohistochemistry, the two antibodies Ki-67 (Kiel 67, antibodies-online GmbH, Aachen, Germany) and α-SMA (alpha smooth muscle actin, Sigma-Aldrich, Darmstadt, Germany) were used. Ki-67 is a proliferation marker used to measure the rate of cell division and proliferation in tissue samples. It is generally used to calculate a Ki-67 proliferation index indicating the rate of increased proliferation in tissue samples. The Ki-67 positive cells are set in relation to all cells within a tissue sample. α-SMA is a marker for smooth muscle cells and myofibroblast activation, which provides an important indication of contraction during wound healing. Each coloration lasted three days and included the following steps:

• Day 1: Tissue deparaffinization, antigen retrieval, blocking with goat serum and overnight incubation with α-SMA and Ki-67 antibodies.
• Day 2: Sections washed, incubated with fluorescent secondary antibodies (Alexa 649 & 488), and 4′,6-Diamidino-2-phenylindol (DAPI) nuclear staining applied (mounted with Mowiol media).
• Day 3: Fluorescence microscopy with specific channels for Ki-67 (red), α-SMA (green), and DAPI (blue) images taken at 40× magnification.

Immunohistochemical analyses were performed with separate evaluation of epidermal and dermal layers to distinguish between incisional skin healing and deeper tissue responses.

Quantitative analysis of immunohistochemical staining was performed using the open-source software ImageJ Version 1.49 (National Institutes of Health [NIH], Bethesda, MD, USA) [28]. Microscopy images were imported in TIFF format, and the mean intensity of α-SMA and Ki-67 staining was measured. Additionally, cell counting was performed for DAPI-stained images. For a more detailed analysis, dermis and epidermis were examined separately, and hair follicles were removed from image processing due to their autofluorescence to avoid interference.

### 2.5. Quantitative Real-Time Polymerase Chain Reaction Analyses of Immunomarkers

For quantitative real-time polymerase chain reaction (qPCR) analysis, RNA was isolated, and complementary DNA (cDNA) was synthesized. For RNA isolation, the QIAGEN RNeasy Mini Kit (Qiagen N.V., Hilden, Germany) was used and performed according to the manufacturer’s protocol. Frozen skin samples were crushed with liquid nitrogen and mixed with a buffer containing beta-Mercaptoethanol (ß-ME) and RNeasy Lysis Buffer (RLT). If needed, mechanical homogenization was carried out using the TissueLyser LT (Qiagen N.V., Hilden, Germany). The samples were centrifuged and the supernatant mixed with ethanol and subsequently pipetted onto columns. After washing steps and drying, RNA was eluted and quantified using a NanoDrop spectrophotometer 2000c (Thermo Scientific™, Waltham, MA, USA) at 260 nm.

cDNA synthesis was performed using the Applied Biosystems™ High-Capacity cDNA Reverse Transcription Kit (Thermo Fisher Scientific, Waltham, MA, USA), following the manufacturer’s protocol. RNA samples were diluted to the lowest concentration, and a master mix was prepared with Reverse Transcription (RT) buffer, deoxyribonucleotide triphosphate mix, random primers, reverse transcriptase, RNase inhibitor, and nuclease-free water. The mix and sample RNA were pipetted into tubes and briefly centrifuged, and cDNA was synthesized using a Thermocycler (FlexCycler2, Analytic Jena, Jena, Germany). Negative controls without reverse transcriptase were included to check for contamination.

Expression levels of the immunomarkers FGF-2, TGF-ß, and VEGF were investigated using qPCR according to standard protocols and manufacturer instructions (Biomol GmbH Hamburg, Germany). Briefly, the gene expression was normalized using β-Actin as a reference. qPCR was performed with SYBR Green in a CFX96™ system (45 cycles, 95 °C/60 °C; Bio-Rad Laboratories Inc., Berkeley, CA, USA). A melting curve analysis verified specificity. Data were analyzed using the ΔΔC_T_-method.

### 2.6. Statistical Analysis

The analysis was performed using GraphPad Prism (Version 10.3.1, GraphPad Software LLC, Boston, MA, USA). Data distribution was assessed graphically and was not consistently normal; therefore, non-parametric statistical methods were applied for group-wise comparisons. Accordingly, results are primarily reported as median with interquartile range (IQR) and, where appropriate, 95% confidence intervals (95%-CI). For comparisons between groups at individual time points (day 14 and day 30), the Kruskal–Wallis test with Dunn’s post hoc test for multiple comparison adjustment was used. For temporal comparisons between day 14 and day 30, a mixed-effects model with Tukey’s post hoc test was applied. This approach was chosen to account for the longitudinal data structure and potential imbalance between groups. The results of the mixed-effects model were interpreted in conjunction with the non-parametric analyses to ensure consistency of findings. An alpha level of 0.05 was considered statistically significant.

This combined approach was used to ensure robust analysis of both cross-sectional and longitudinal effects in the presence of non-normally distributed data.

## 3. Results

Four interventional groups were differentiated: Sham (no hemostatic agent applied), ONRC (Resorba^®^ CELL), ORC (Tabotamp^®^), and GELA (GELITA-TUFT-IT^®^). Investigated time points after surgical intervention were 14 and 30 days. The selection of the investigated hemostatic biomaterials was based on their widespread clinical use and differing structural and biochemical properties, which may influence wound-healing outcomes. The time points were chosen to coincide with the early and late phases of the remodeling process, which typically begins two to three weeks after injury and is critical for the resolution of inflammation, ECM maturation, and scar formation [9]. All findings must be interpreted within the context of a primary intention incisional wound-healing model with subfascial biomaterial implantation. As the epidermis was surgically closed, the presented results reflect dermal and subcutaneous tissue responses and remodeling processes rather than open wound closure or re-epithelialization dynamics typically observed in excisional wound models.

**Figure 1:**
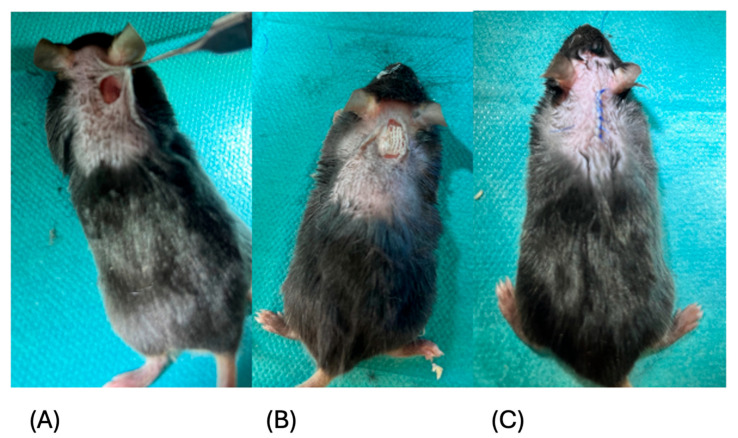
Representative visualization of the experimental setup. (**A**–**C**) Photographic documentation of the surgical procedure at the time of surgery (day 0): (1) after incision, (2) after application of the hemostatic agent, and (3) after wound closure using sutures.

Figure 1 shows representative photographic documentation of the surgical procedure at day 0 (panels A–C).

### 3.1. Hematoxylin-Eosin and Collagen (Movat Pentachrome) Staining

All samples underwent HE and collagen (MP) staining with qualitative microscopic evaluation to characterize tissue morphology and structural wound features.

Histological findings are presented as qualitative observations, as no standardized histomorphometric or semi-quantitative scoring system was applied.

**Figure 2:**
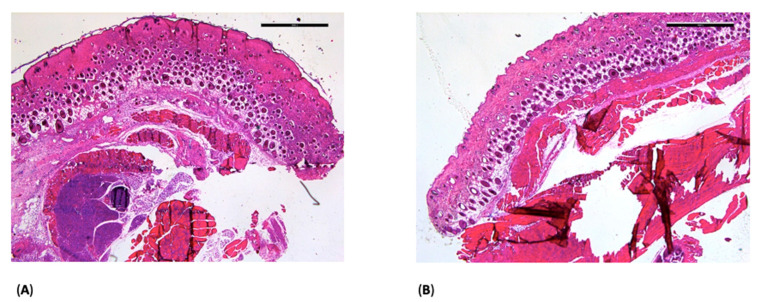
Light microscopy images of the ORC (**A**) and ONRC groups (**B**) at day 14 after HE staining; 2.5× magnification (scale bar: 1 mm). No hemostatic agent residues are visible.

At postoperative day 14, HE staining showed normal tissue morphology in the ORC (Figure 2A) and ONRC groups (Figure 2B), with no detectable hemostatic agent residues in the analyzed sections.

**Figure 3:**
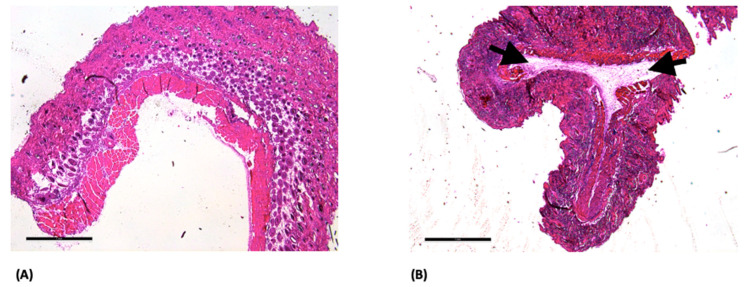
Light microscopy images of Sham (**A**) and GELA (**B**) groups at day 14 after HE staining; 2.5× magnification (scale bar: 1 mm). In (**B**), the arrows (→) mark a fibrous structure, which most likely represents yet undegraded gelatinous parts of the material embedded during surgery.

At postoperative day 14, HE staining of the Sham group demonstrated tissue architecture without apparent structural irregularities based on qualitative evaluation. In contrast, the GELA group exhibited a localized fibrous structure at the implantation site (Figure 3B), which was not observed in the Sham control (Figure 3A). This structure may represent residual or partially degraded material; however, this interpretation is based on qualitative assessment and cannot be confirmed without additional material-specific analyses.

**Figure 4:**
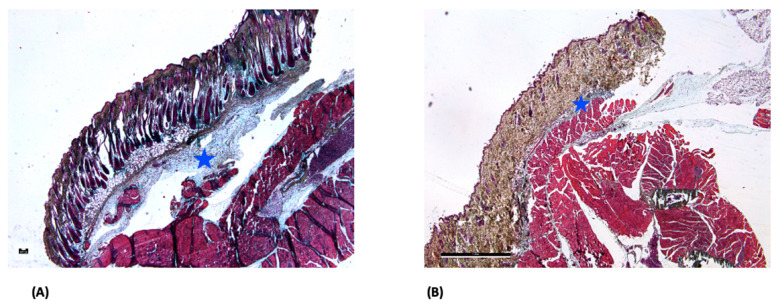
Light microscopy images of GELA group at days 14 (**A**) and 30 (**B**) after MP staining; 2.5× magnification (scale bar: 50 µm (**A**) and 1 mm (**B**)). In both figures, the star (☆) marks the blue-stained area, representing increased newly formed ECM.

MP staining of the GELA group demonstrated visually increased blue-stained ECM structures at both postoperative days 14 and 30 (Figure 4). Compared with other groups, this suggests a higher degree of ECM deposition; however, this observation is based on qualitative histological assessment without quantitative morphometric validation.

**Figure 5:**
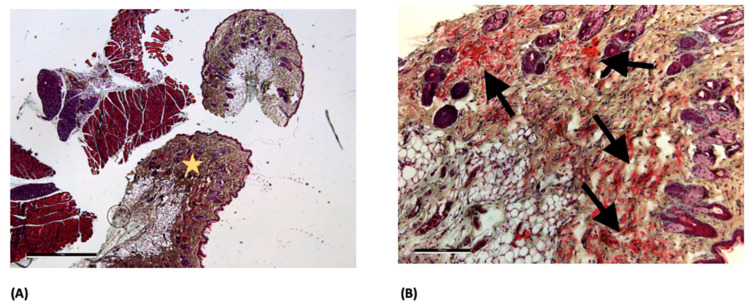
Light microscope images of the OCR group at day 30 after MP staining. (**A**) 2.5× magnification (scale bar: 1 mm) and (**B**) 10× magnification (scale bar: 200 µm). The magnified area is marked with a star (☆) in image (**A**). In image (**B**), the arrows (→) mark pronounced streaks of red fibers, which potentially represent an increased myofibroblast deposition and activity.

Pronounced red-stained fiber structures were observed in the ORC group at postoperative day 30 (Figure 5). These structures may indicate connective tissue organization; however, this interpretation remains descriptive and cannot be quantitatively confirmed. The observed pattern could potentially be associated with myofibroblast activity, although no direct cell-specific analysis was performed to support this assumption.

### 3.2. Ki-67 Activity (Proliferation)

**Figure 6:**
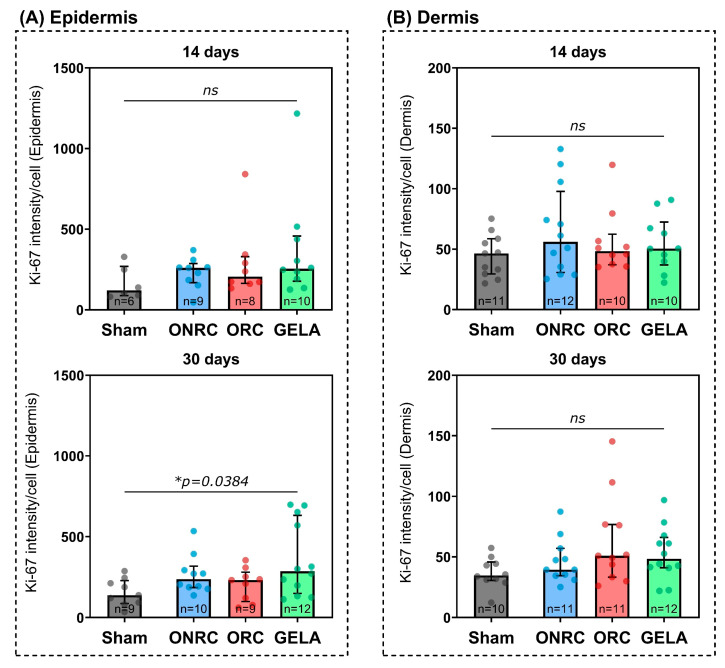
Results of the immunohistochemical staining of the marker Ki-67 in the epidermis (**A**) and dermis (**B**) after 14 and 30 days (upper and lower graphs, respectively). A significant difference was observed only for GELA compared to Sham in the epidermis after 30 days. * *p* < 0.05. ns: not significant.

No significant differences were found between the investigated groups regarding Ki-67 intensity after 14 days (Figure 6). In the epidermis, ONRC (260.1, 95%-CI [152.6; 309.7]) and GELA (255.2, 95%-CI [135.3; 515.7]) demonstrated the highest intensity, while Sham (120.9, 95%-CI [81.4; 328.4]) showed the lowest. ORC demonstrated a lower intensity than other hemostats yet still elevated compared to the untreated Sham group (205.8, 95%-CI [133.6; 841.5]). The same distribution was observed in the dermis but with an overall lower Ki-67 intensity for all groups. ONRC (56.1, 95%-CI [29.2; 105.7]) and GELA (50.5, 95%-CI [28.1; 87.7]) demonstrated the highest intensity, while ORC showed a lower intensity than the other hemostats (48.4, 95%-CI [35.7; 79.5]). The Sham group again demonstrated the lowest intensity of all groups.

At the 30-day time point, GELA (286.5, 95%-CI [133.0; 652.8]) significantly (*p* = 0.0384) increased Ki-67 activity compared to the Sham group (138.0, 95%-CI [81.0; 245.0]) in epidermal cells (Figure 6A). ONRC and ORC did not demonstrate a significant increase in Ki-67 activity, either in the epidermis or in the dermis. In the dermis, GELA also showed no significant increase in Ki-67 activity compared to Sham, while ORC demonstrated the overall highest activity in the dermis after 30 days (50.9, 95%-CI [30.0; 111.6]; Figure 6B).

**Figure 7:**
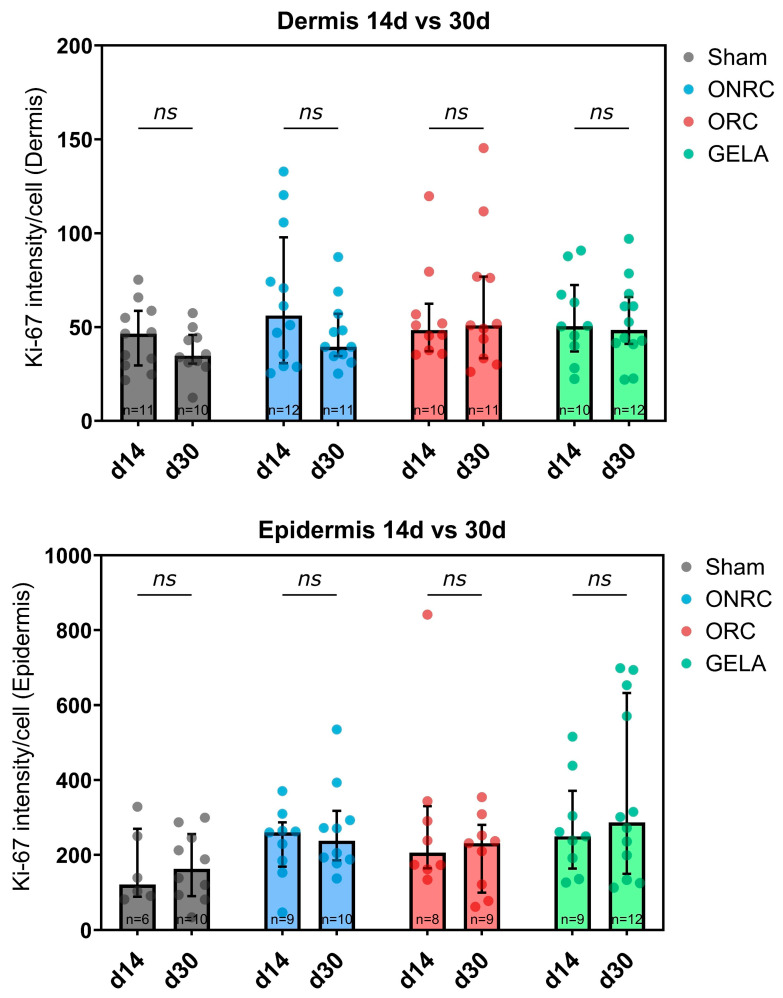
Levels of Ki-67 activity in the dermis (**upper graph**) and epidermis (**lower graph**) between days 14 and 30 by group. No significant difference was observed between the time points for any group. ns: not significant.

Differences in Ki-67 activity between days 14 and 30 were also investigated to determine temporal trends (Figure 7). While in the dermis Ki-67 activity decreased between days 14 and 30 for all investigated groups except ORC, the opposite was observed in the epidermis, where Ki-67 intensity showed higher levels at day 30 for all groups except for ONRC. However, none of these comparisons showed a statistically significant difference.

### 3.3. α-SMA Activity (Contraction)

**Figure 8:**
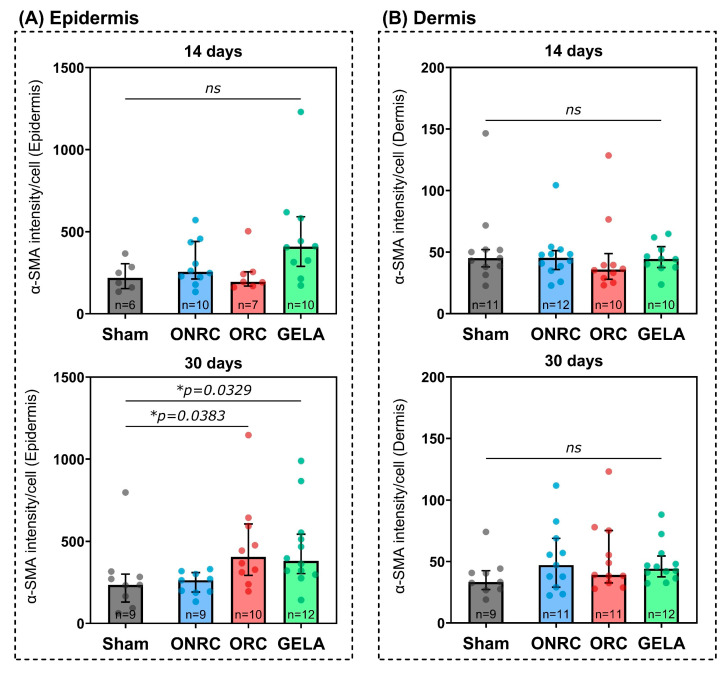
Changes in α-SMA activity in the epidermis (**A**) and dermis (**B**) after 14 and 30 days of hemostat (ORC, ONRC, and GELA) application compared to Sham. After 30 days, α-SMA expression in the epidermis was significantly higher after the use of ORC (*p* = 0.0383) and GELA (*p* = 0.0329) compared to the Sham group. * *p* < 0.05.

For α-SMA, no significant differences between the groups were observed after 14 days, either for the epidermis (Figure 8A) or for the dermis (Figure 8B). GELA demonstrated the highest α-SMA intensity (408.5, 95%-CI [213.7; 618.8]) compared to Sham (218.7, 95%-CI [134.3; 367.0]) in the epidermis after 14 days, while ONRC showed the highest intensity in the dermis (45.4, 95%-CI [35.0; 52.1]) but only slightly higher than the Sham group, which showed the second highest intensity (45.1, 95%-CI [31.5; 71.6]).

After 30 days, both ORC (405.2, 95%-CI [238.8; 643.9]; *p* = 0.0383) and GELA (380.7, 95%-CI [297.8; 553.5]; *p* = 0.0329) demonstrated significantly elevated intensity levels for α-SMA in the epidermis compared to the Sham group (235.0, 95%-CI [93.1; 316.6]). In the dermis, however, no significant differences were observed, and ONRC actually demonstrated the highest α-SMA intensity compared to the Sham group (47.1, 95%-CI [23.6; 82.7] vs. 33.4, 95%-CI [26.8; 44.2]).

**Figure 9:**
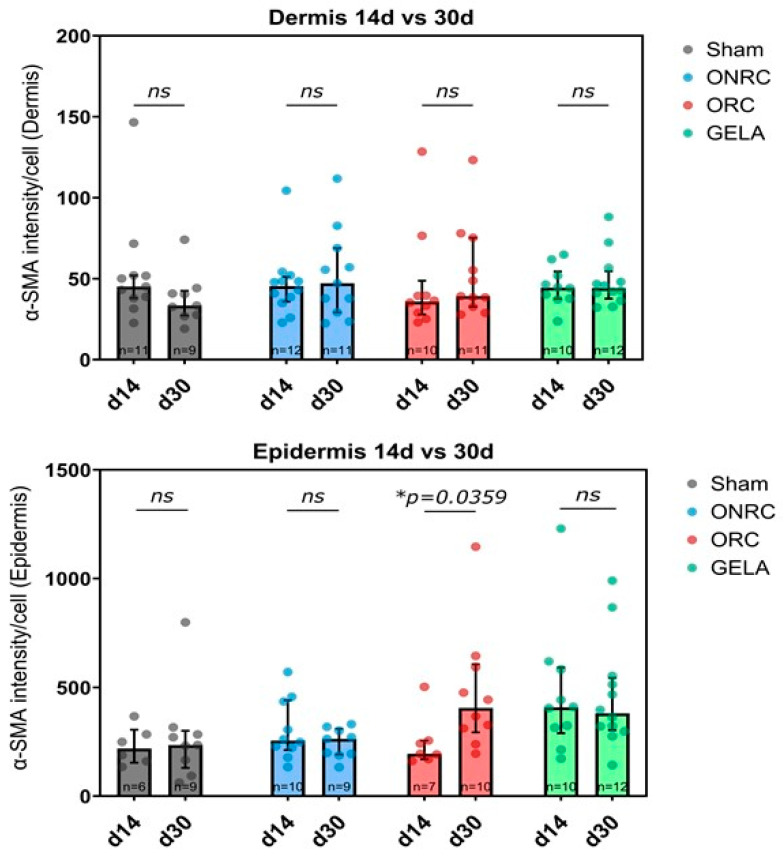
Time-dependent changes in α-SMA expression in the dermis (**upper graph**) and epidermis (**lower graph**). A significant increase in α-SMA activity was observed in epidermal cells after using ORC between 14 and 30 days (Δ = 228.1, 95%-CI [16.2; 440.1]; *p* = 0.0359). * *p* < 0.05. ns: not significant.

Moreover, a time-dependent increase in α-SMA activity in epidermal cells was found for ORC between 14 and 30 days (Δ = 228.1, 95%-CI [16.2; 440.1]; *p* = 0.0359; Figure 9).

### 3.4. Growth Factors

To quantify differences in growth factor expression between the four groups, a real-time qPCR analysis was performed. The markers VEGF, TGF-ß, and FGF-2 were evaluated.

### 3.5. VEGF

**Figure 10:**
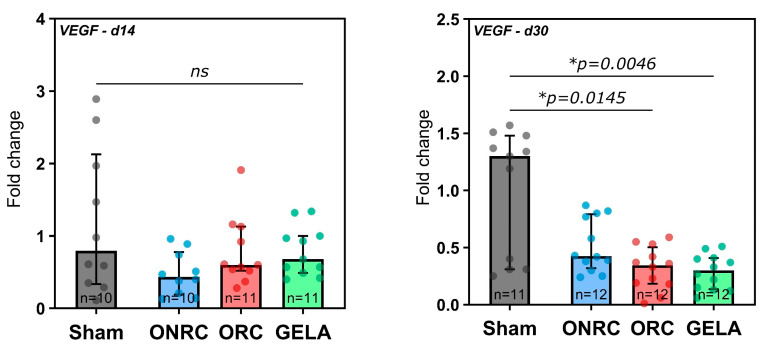
Fold changes in VEGF expression after application of ONRC, ORC, and GELA compared to Sham at 14 and 30 days. After 30 days, a significantly lower VEGF expression was observed for ORC (*p* = 0.0145) and GELA (*p* = 0.0046). * *p* < 0.05. ns: not significant.

In terms of VEGF expression, no significant differences were found between the groups 14 days post-surgery (Figure 10). However, at 30 days VEGF production was significantly inhibited by GELA (0.30, 95%-CI [0.19; 0.38], *p* = 0.0046) and ORC (0.34, 95%-CI [0.19; 0.43], *p* = 0.0145) compared to the Sham group (1.30, 95%-CI [0.31; 1.51]; Figure 10).

**Figure 11:**
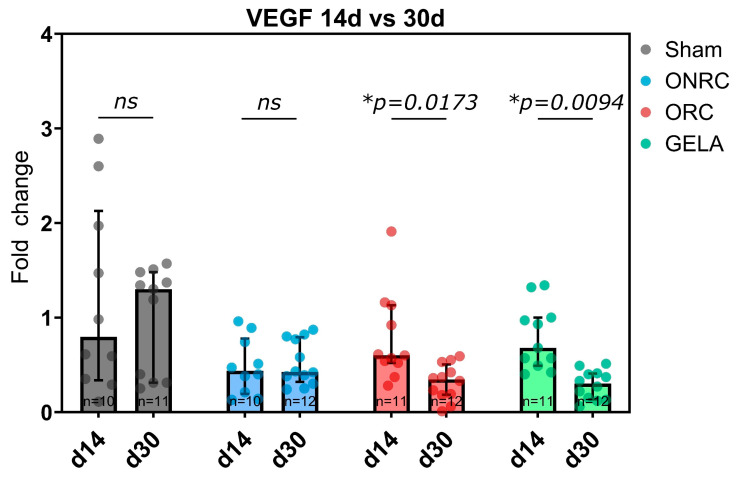
Time-dependent changes in VEGF expression between days 14 and 30 for each group. A significant reduction in expression was observed in the ORC (*p* = 0.0173) and GELA groups (*p* = 0.0094) between the time points. * *p* < 0.05. ns: not significant.

When comparing the 14-day and 30-day results for each group, we observed that VEGF expression was significantly reduced over time after ORC (Δ = 0.45, 95%-CI [0.08; 0.82], *p* = 0.0173) and GELA application (Δ = 0.50, 95%-CI [0.13; 0.87], *p* = 0.0094; Figure 11).

### 3.6. TGF-ß

**Figure 12:**
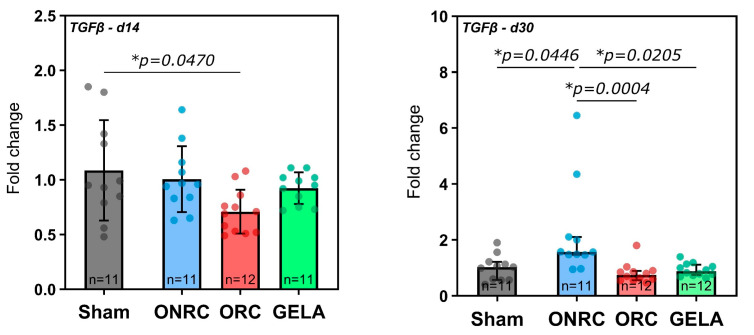
Fold changes in TGF-β expression after application of ONRC, ORC, and GELA compared to Sham at 14 and 30 days. After 14 days, ORC was significantly downregulated compared to Sham (*p* = 0.0470), while after 30 days, ONRC was significantly upregulated compared to Sham (*p* = 0.0446). ORC and GELA showed significantly lower expression compared to ONRC after 30 days (*p* = 0.0004 and *p* = 0.0205, respectively). * *p* < 0.05. ns: not significant.

After 14 days, TGF-ß expression was significantly inhibited by ORC (0.70, 95%-CI [0.52; 0.86], *p* = 0.0470) compared to the Sham group (0.95, 95%-CI [0.56; 1.8]) but not significantly affected by using ONRC or GELA (Figure 12). Conversely, after 30 days, TGF-β expression was significantly higher in the ONRC group (1.57, 95%-CI [1.09; 3.34]) in comparison to the Sham group (1.03, 95%-CI [0.69; 1.31], *p* = 0.0446). TGF-β expression in ORC (0.75, 95%-CI [0.58; 1.03]) and GELA (0.88, 95%-CI [0.78; 1.07]) was significantly lower than ONRC (*p* = 0.0004 and *p* = 0.0205, respectively; Figure 12).

**Figure 13:**
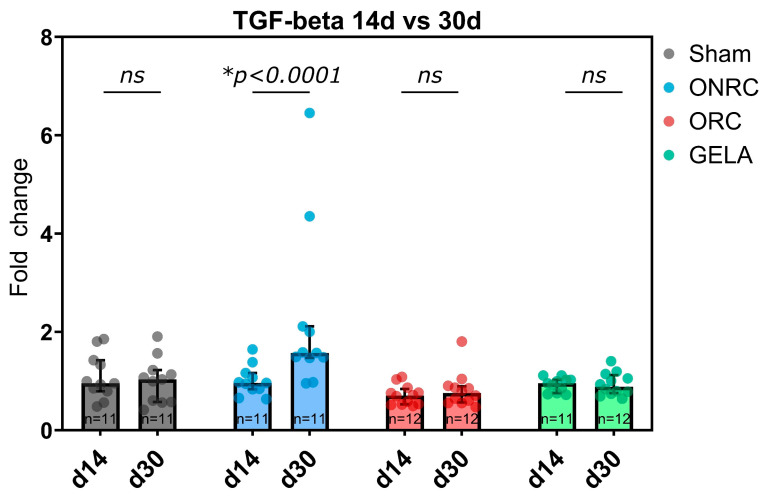
Time-dependent changes in TGF-β expression between days 14 and 30 for each group. A significant increase in expression was observed in the ONRC group (*p* < 0.0001) between the time points. * *p* < 0.05. ns: not significant.

In regard to changes within the groups between days 14 and 30, TGF-β expression increased significantly over time in the ONRC group (Δ = 1.21, 95%-CI [0.67; 1.76], *p* < 0.0001), while no significant changes between time points were observed for the other groups (Figure 13).

### 3.7. FGF-2

**Figure 14:**
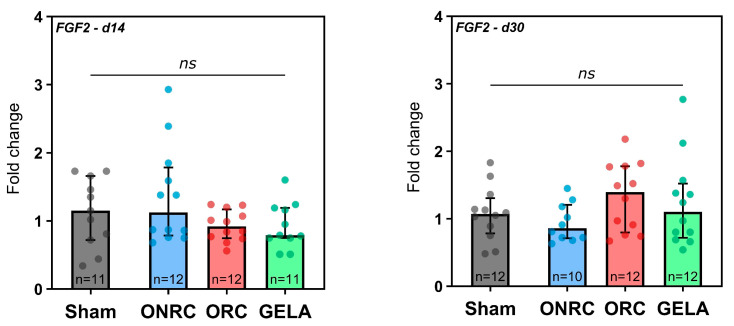
Fold changes in FGF-2 expression after application of ONRC, ORC, and GELA compared to Sham at 14 and 30 days. No significant differences were observed in FGF-2 expression after hemostat application. * *p* < 0.05. ns: not significant.

No significant differences in FGF-2 levels were observed between the Sham group and the investigated hemostats, either at 14 days or at 30 days post-surgery (Figure 14). While all hemostats showed lower median expression levels at 14 days compared to the Sham group, GELA (1.07, 95%-CI [0.69; 1.57]) showed an equal level after 30 days and ORC (1.30, 95%-CI [0.76; 1.78]) even displayed a higher level compared to the Sham group.

**Figure 15:**
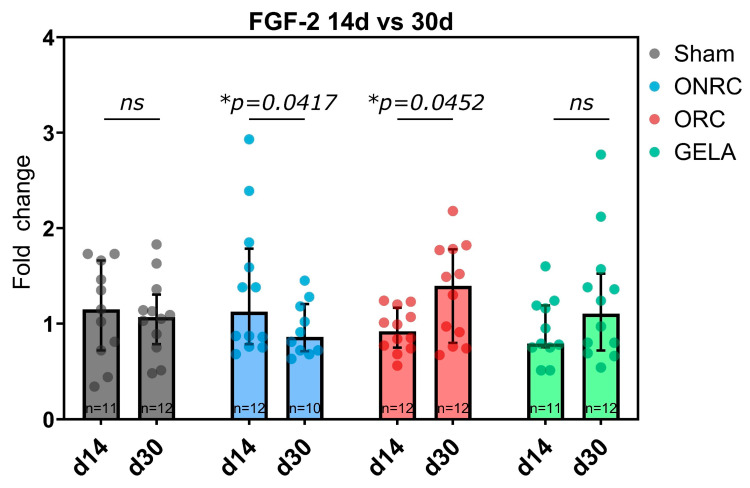
Time-dependent Time-dependent changes in FGF-2 expression between days 14 and 30 for each group. ONRC significantly inhibited FGF-2 expression over time (*p* = 0.0417), whereas ORC significantly increased (*p* = 0.0452) FGF-2 expression. * *p* < 0.05. ns: not significant.

However, time-dependent changes in FGF-2 expression were observed within the individual hemostat groups (Figure 15). For ONRC, a reduction in expression level was observed between days 14 and 30 (Δ = −0.42, 95%-CI [−0.83; −0.01]; *p* = 0.0417), while ORC induced an increase in FGF-2 expression over time (Δ = 0.39, 95%-CI [0.009; 0.78], *p* = 0.0452).

## 4. Discussion

An important factor in the interpretation of the findings is the specific wound model used, which in this case represents a primary intention incisional healing scenario with subfascial implantation of hemostatic biomaterials. In this setting, the epidermis is closed immediately after surgery, and healing primarily occurs within the dermal and subcutaneous tissue layers. This is fundamentally different from excisional or secondary intention wound models, where wound closure dynamics, granulation tissue formation, and re-epithelialization across an open wound surface are the primary outcome measures. The results should therefore be interpreted as reflecting tissue remodeling quality and biomaterial–tissue interaction beneath a closed incision, rather than macroscopic wound closure kinetics.

HE staining showed no obvious structural abnormalities in the ORC and ONRC groups (Figure 2), based on qualitative histological assessment. In contrast, HE staining revealed localized fibrous structures in the GELA group unlike in the Sham control at the same time point (Figure 3). These findings may indicate enhanced connective tissue formation during wound healing following GELA application.

In the late phase of wound healing, collagen deposition represents a key regulator of regenerative pathways and overall wound quality [29]. Collagen can interact with different regenerative pathways in skin wound healing [30]. Consistent with this physiological role, ME staining demonstrated more pronounced blue collagen deposition in the GELA group (Figure 4), suggesting enhanced ECM formation during the late proliferative and remodeling phases of wound healing. The presence of localized fibrous structures and increased ECM deposition in the GELA group at day 14 raises the possibility of downstream effects on long-term scar architecture and mechanics. While augmented matrix formation may enhance early tissue stability, its persistence could also predispose to increased scar stiffness or fibrotic remodeling. However, interpretation is limited by the 30-day observation window and the absence of functional or biomechanical assessment. Consequently, no conclusions can be drawn regarding the mechanical properties of the resulting scar tissue. Future studies incorporating extended follow-up and quantitative biomechanical testing (e.g., tensile strength and elasticity) are required to determine whether these matrix alterations translate into clinically relevant differences in scar quality. The concomitant increase in Ki-67 expression (Figure 6) supports the notion of sustained cellular activity accompanying matrix deposition. This is consistent with previous experimental data suggesting that gelatin-based biomaterials may promote proliferative responses in parallel with ECM formation. For example, a study in diabetic mice reported significant beneficial effects of photo-crosslinked gelatin combined with bFGF on wound closure, including increased fibroblast numbers, augmented collagen production, and enhanced granulation tissue formation compared with bFGF alone [31]. Increased collagen deposition may contribute positively to wound stability by strengthening the ECM; however, excessive matrix formation has also been associated with fibrotic healing responses. Notably, no clinical signs of excessive scarring were observed in the GELA-treated animals in the present study, suggesting that the enhanced matrix formation remained within a physiologically adaptive range.

The effect of hemostatic biomaterials on fibroblast proliferation has been previously investigated in several pre-clinical studies [32,33,34], which reported outcomes to be dependent on the hemostatic material used. Ki-67 has been previously used to assess fibroblast proliferation, for example in hypertrophic scar tissue, via immunofluorescence [35]. Ki-67 plays a key role in cell-cycle regulation and mitosis [21], only present in “active phases of the cell cycle,” peaking in early mitosis [20]. In the clinical context, Ki-67 is used to assess the prognosis of various tumor types as a marker for cancer cell proliferation [19,20].

In our study, a similar dependence on the tested material in terms of proliferation was observed for the gelatin-based hemostat. GELA was significantly associated with increased cell proliferation in epidermal cells after 30 days of treatment, as indicated by an increase in Ki-67 intensity (Figure 6). The prolonged proliferative activity observed in the GELA group at day 30 may be related to its longer in vivo residence time compared to cellulose-based materials. While ORC and ONRC are reported to be largely resorbed within approximately 14 days, gelatin-based materials such as GELA may persist for up to 30 days, as also suggested by the continued presence of ECM–like structures in histological staining.

This extended presence may provide a sustained structural scaffold or microenvironment that supports ongoing cellular activity, including fibroblast proliferation and matrix production. In this context, the material may act not only as a hemostatic agent, but also as a temporary matrix influencing local tissue remodeling dynamics. However, as no direct measurements of material degradation or local cellular interactions were performed, this association remains speculative. Further studies combining degradation kinetics with cell-specific analyses would be necessary to establish a causal relationship.

Previous in vitro and in vivo studies have demonstrated that gelatin-based materials can generally stimulate the proliferation of fibroblasts [36]. Notably, our previous in vivo study on the early effects of hemostatic biomaterials confirmed this proliferative effect at earlier time points, further supporting the sustained pro-proliferative properties of gelatin-based hemostats [27]. In another study, gelatin was shown to stimulate embryonic mouse fibroblasts in vitro [37], and another reported that a gelatin- and alginate-based hydrogel enhanced cell viability and proliferation in mouse fibroblasts after 7 and 14 days [38]. Furthermore, an alginate hydrogel containing gelatin exhibited immediate positive effects on cell viability and proliferation in normal human dermal fibroblasts, compared to an alginate-only hydrogel [39]. Testing of a gelatin-containing wound dressing on porcine skin similarly showed increased fibroblast proliferation [40]. Our results are in line with these findings, indicating a positive effect of gelatin-based hemostats on proliferation. GELA already demonstrated a higher intensity after 14 days with significantly higher activity of Ki-67 after 30 days.

Current studies on the influence of oxidized cellulose (regenerated as well as non-regenerated) on cell proliferation are limited and contradictory. Rassu et al. demonstrated that the use of ORC in oncoplastic breast surgery could enhance dermal fibroblast proliferation and cell migration, leading to positive outcomes for shape adjustment [41]. In contrast, Krishnan et al. reported that ORC had a negative impact on wound healing in vivo and found no beneficial effects on cell proliferation or migration [42]. Other results were obtained in a study investigating the relationship between ORC and mucosal healing in an experimental animal model, with no significant influence of ORC on proliferation reported [43]. Another study conducted by Wagenhäuser et al. within our department found that both ORC and ONRC inhibit fibroblast proliferation and migration in vitro [23]. In light of the contradictory results, it is impossible at this time to make a definitive statement about the effect of oxidized cellulose on fibroblast proliferation in wound healing. Our current study demonstrated no stimulating effect of ORC or ONRC on dermal or epidermal fibroblast proliferation within 30 days of treatment. In contrast to earlier reports, however, our experiments in the mouse model also showed no inhibition of proliferation, with overall similar Ki-67 levels compared to the Sham group. We can therefore conclude that, while ORC or ONRC treatment yields no benefit, no harm is done to the regenerative process.

In contrast to cellulose-based materials, gelatin represents a biologically derived matrix with distinct physicochemical and degradation properties. Its prolonged presence in the tissue may provide a temporary scaffold that supports sustained cellular activity, including proliferation and ECM production. This highlights the importance of both material composition and persistence in shaping tissue remodeling responses.

Another important indicator of fibroblast activity, especially in terms of migration and myofibroblast activity, is α-SMA. The central role of α-SMA in the control of (myo)fibroblasts has been widely emphasized in the literature [16,17,44]. In a porcine burn model, a direct association of in vivo burn scar contraction with the level of α-SMA present in scar tissue was observed: at week 2 after burn, the level of α-SMA expression in 16 burn wounds was significantly related to the depth of burn and wound-healing outcome [45]. Another experimental animal study investigated the effect of oxidized regenerated cellulose on the healing of pharyngeal wounds and showed no significant difference in α-SMA expression level between wounds treated with and without ORC [43].

In our study, α-SMA expression was significantly activated during treatment with ORC and GELA after 30 days (Figure 8), while ONRC showed no significant changes in α-SMA expression compared to the control Sham group. Again, this was only true for epidermal cells, whereas dermal cells showed no significant effects for any hemostat treatment. The fact that significant effects were only observed for epidermal cells might be due to the form of wound healing, which can be characterized as primary healing, where wound edges are adapted using sutures. Therefore, primarily the epidermal gap needs to be bridged by proliferation and contraction, while in secondary wound healing actual tissue regeneration on a dermal level with restructuring of a wound bed for proper epithelization would be necessary. In secondary healing, more dermal activity might be observed. The absence of inhibition of α-SMA expression following the application of the various hemostats suggests that this may account for the lack of wound contraction issues observed in the mice in our study.

Contrary to the findings of our current study, a previously performed in vitro study from our department found that ORC and ONRC inhibited fibroblast-mediated matrix contraction [23]. This could not be replicated in the mouse model and rather showed a time-dependent increase in α-SMA expression as an indicator of increased contraction and fibroblast migration, at least for ORC after 30 days. The time-dependent aspect was highlighted by the significant increase in α-SMA expression between 14 and 30 days, which is in line with wound contraction being primarily part of the later epithelization and re-organizational phase of wound healing. The contradictory results might be an expression of the differing source organisms, with human in vitro cells in a monoculture demonstrating a reduced capability of contraction compared to the complex tissue situation in the mouse model.

The impact of gelatin on wound contraction remains debated, with studies reporting varying results; however, a growing body of research indicates a positive effect on wound contraction. For instance, an in vitro and in vivo study in Wistar rats found accelerated skin wound contraction when treated with a combination of gelatin-nanofibers and polyvinyl alcohol hydrogel, compared to controls without wound dressing [46]. Another in vitro study demonstrated enhanced contraction of rat cardiac myocytes following the application of a gelatin-containing hydrogel [47]. Furthermore, an in vivo study on Göttingen minipigs confirmed the beneficial effect of gelatin-collagen materials on wound contraction [48]. A combination of gelatin and chitosan also showed a positive impact on wound contraction after 14 days in a separate in vivo study in Wistar rats [49]. Our findings indicate that the gelatin-based hemostat was associated with increased α-SMA expression after 30 days in epidermal cells. As α-SMA serves as a surrogate marker of contraction-related activity, this observation may reflect altered cellular remodeling processes rather than direct functional contraction. Similarly, based on Ki-67 and α-SMA expression, GELA was associated with increased cellular activity at the epidermal level.

To further investigate the impact of the various tested hemostats on a biomolecular level in terms of key regulators in relevant processes such as angiogenesis, fibrosis, and tissue regeneration, VEGF, TGF-β, and FGF-2 levels were analyzed. VEGF stimulates and increases neovascularization [50] and plays a vital role in angiogenesis by stimulating the proliferation of postcapillary endothelial cells through nitrogen oxide production and cyclic guanosine monophosphate accumulation, which are critical for the development of well-perfused granulation tissue [12,51]. An in vivo experiment on pig skin demonstrated promising wound-healing outcomes, including enhanced angiogenesis and collagen deposition, achieved through the application of a 3D-printed gelatin hydrogel patch combined with a VEGF-mimicking peptide [52]. Another recent in vivo study involving VEGF-overexpressing fibroblasts in mice showed a reduction in wound area, enhanced angiogenesis, and the formation of granulation tissue, highlighting the important role of VEGF in promoting wound healing [53]. In a previous in vitro study conducted by our research group, we observed that GELA induces VEGF expression after 24 h [4]. In contrast, investigation in the current study of the later time points in the reparative process revealed a significant inhibition of VEGF expression in ORC and GELA after 30 days compared to Sham. After 14 days, in contrast, no significant difference between groups was observed. Based on these findings, it can be inferred that if wound healing is completed within the first two weeks, the subsequent effect of ORC and GELA on VEGF expression may be of limited relevance and reflect a normal resolution of the angiogenic response as the wound transitions into the late remodeling phase, during which neovascularization is no longer actively required [54,55]. The observed reduction in VEGF expression at day 30 in the ORC and GELA groups may reflect a transition into a later remodeling phase with reduced angiogenic activity. However, this interpretation remains speculative and cannot be confirmed by the present data alone. Alternative explanations must be considered. Lower VEGF expression may also indicate reduced or insufficient angiogenic activity, differences in tissue composition, or normal biological variability during the remodeling phase. As gene expression analysis was performed on bulk tissue samples, it is not possible to determine whether the observed changes were due to differences in cell number, altered expression per cell, or shifts in cellular composition within the wound tissue. The observed VEGF dynamics should therefore be interpreted cautiously and considered indicative rather than definitive for the underlying biological processes. Taken together, these results highlight the limitations of interpreting angiogenic activity solely based on bulk gene expression data at isolated time points.

Although day 30 does not represent end-stage fibrosis, it constitutes a clinically relevant postoperative remodeling phase in which wound healing either proceeds toward physiological maturation or shifts toward pathological outcomes such as excessive fibrosis, hypertrophic scarring, or delayed healing. Therefore, early regulatory markers of remodeling, including TGF-β, α-SMA, VEGF, and FGF-2, were analyzed to assess the direction of the healing process rather than established fibrosis. Later time points were not included, as the primary aim was to capture this decisive early remodeling phase rather than late scar consolidation.

Similar observations in terms of marker dynamics over time were made for TGF-β in this study. ORC demonstrated significantly lower levels of TGF-β at 14 days compared to the Sham group, and generally all hemostats showed lower levels than Sham. The only exception was ONRC after 30 days, which demonstrated a significantly higher TGF-β expression not only than ORC and GELA, but also than the Sham group (Figure 12), with a significant increase between days 14 and 30 (Figure 13). The observed increase in TGF-β expression in the ONRC group at day 30 may indicate a temporal shift in the remodeling phase compared to the other materials. One possible interpretation is that non-regenerated cellulose induces a delayed peak in TGF-β-mediated signaling, suggesting a prolonged remodeling response. This may reflect differences in material structure and degradation behavior, potentially leading to sustained cellular activation or prolonged inflammatory signaling. In contrast, the earlier normalization of TGF-β levels in ORC and GELA may indicate a more rapid transition through the remodeling phase. However, this interpretation remains hypothetical, as the present study does not include intermediate time points or direct assessment of inflammatory kinetics. Additional temporal analyses would be required to confirm whether ONRC indeed induces a delayed remodeling trajectory. Despite their chemical similarity, ORC and ONRC differ in their physical structure, fiber organization, and degradation characteristics. Regenerated cellulose forms more organized fiber structures, whereas non-regenerated cellulose exhibits a more heterogeneous and less structured architecture. These structural differences may influence cellular infiltration, degradation kinetics, and local microenvironmental conditions, ultimately leading to distinct temporal patterns of cytokine and growth factor expression. Accordingly, the early inhibition of TGF-β in ORC at day 14, followed by normalization, may reflect a more rapid resolution of the inflammatory phase, whereas the delayed increase observed in ONRC could indicate prolonged tissue interaction or slower degradation. Overall, these findings suggest that differences in tissue response are driven not only by chemical composition, but also by material structure and organization. Nevertheless, the underlying mechanisms remain unclear and require further investigation.

The impact of hemostats on TGF-ß, as an essential inflammatory and reparative regulator in wound healing, is of particular interest. The critical role of TGF-β in physiological wound healing was highlighted in an earlier study that used a mouse model with fibroblast-specific deletion of the type II TGF-β (TβRII) receptor. The study showed that deletion of TβRII in fibroblasts impaired normal wound healing. Specifically, the authors found that TGF-β signaling in fibroblasts is essential for regulating ECM production, granulation tissue formation, and proper wound contraction. The absence of this signaling led to defective wound healing, highlighting the pivotal role of TGF-β in coordinating these processes during tissue repair [56]. One of our recent in vitro studies found that the application of ORC, ONRC, and GELA does not affect the expression of TGF-ß in the early wound-healing response after 24 h [8]. However, in light of our results, this might be due to the early time point of 24 h, as TGF-β mainly regulates later parts of the inflammatory response and orchestrates processes of fibrosis and fibroplasia as a main regulator [57]. TGF-β has also been observed to function as a driver in prolonged and dysregulated inflammatory responses during the healing process, which can ultimately result in healing disorders and prolonged overall healing duration [58]. While its transient expression is essential for normal tissue repair, sustained or excessive TGF-β signaling during the remodeling phase—which typically begins two to three weeks after injury—has been strongly associated with fibrotic tissue deposition and pathological scarring [59,60]. Prolonged TGF-β activity stimulates the differentiation of fibroblasts into myofibroblasts, enhances collagen type I synthesis, and inhibits matrix metalloproteinases, thus reducing ECM turnover [61,62]. These processes contribute to persistent fibrosis, particularly when tissue remodeling fails to resolve properly. Therefore, an increase in TGF-β expression over time may indicate a profibrotic tendency at the molecular level. Assessing TGF-β expression in response to different hemostatic agents thus offers valuable insight into their potential to influence long-term wound remodeling and scar formation. Moreover TGF-β acts as a suppressor of re-epithelialization during wound healing. In murine models, re-epithelialization was significantly accelerated when a dominant-negative TGF-β receptor was expressed specifically in the epidermis [63]. The observed inhibition of TGF-ß by ORC after 14 days and generally lower levels observed for all hemostats might therefore indicate an attenuation of the TGF-β-induced response in the healing process, which can be beneficial in terms of preventing excessive inflammation. Additionally, the increased stimulation of TGF-ß by ONRC may in turn have a negative effect on wound healing, inducing extensive ECM deposition, contraction, and therefore fibrosis-induced scarring of the wound [57,64].

Although the acute control of bleeding often takes precedence over long-term outcomes, awareness of these molecular effects is crucial. A better understanding of how different hemostats influence TGF-β activity and downstream fibrogenesis could inform more tailored clinical decision-making, especially in surgeries where minimizing scarring is a priority or in patients predisposed to excessive fibrosis.

However, these interpretations should be made with caution, as the present data do not allow direct conclusions regarding functional fibrosis or clinical scar formation.

FGF-2 represents another highly relevant effector in the reparative process. FGF-2 is known to enhance collagen synthesis, wound contraction, epithelialization, and the production of fibronectin and proteoglycans [13,65]. In our animal study, however, no influence of the investigated hemostats on FGF-2 was observed at 14 or 30 days compared to the Sham group. However, an interesting opposing effect of ONRC and ORC was observed. While ONRC demonstrated a significant decrease in FGF-2 expression between days 14 and 30, which is in line with the temporal changes observed in the Sham group, the ORC group demonstrated a significant rise in FGF-2 activity (Figure 15). An increase over time was also observed for GELA, but it was not statistically significant.

There is limited research on the direct effect of oxidized cellulose on FGF. An in vitro study explored the inhibitory effects of ORC and ONRC on key wound-healing processes, which may indirectly influence the activity of growth factors such as FGF and therefore fibroblast activity [23]. Another study examined novel cellulose derivatives and their mitogenic activity in the presence and absence of FGF-2. Although the study did not directly address oxidized cellulose, it provides insights into the interactions between cellulose derivatives and FGF [66]. The effect of gelatin on FGF has been studied more extensively. Suzuki et al. demonstrated that using gelatin gel as a carrier for growth factors such as TGF-ß and FGF-2 significantly improved angiogenesis and granulation tissue development [67]. In a study by Kawai et al., an artificial dermis model revealed that FGF-2-impregnated gelatin microspheres promoted greater fibroblast proliferation and tissue regeneration compared to free FGF-2 [68]. Furthermore, Jinno et al. (2016) observed that rats treated with an FGF-2-impregnated gelatin/collagen sponge showed significantly better development of skin-like tissue compared to those treated with a collagen sponge alone [69]. A more recent study investigated the adipogenic properties of a collagen/gelatin sponge releasing FGF-2 in the subcutis of mice, identifying the material as a promising approach for treating soft tissue defects [70]. The inhibition of FGF-2 by ONRC observed in the present study could therefore be detrimental to wound healing. In contrast, the significant increase in FGF-2 activity in the ORC group and similar tendencies in the GELA group can be construed as positive for the healing process and can improve overall healing.

Several previous studies have described a positive effect on wound healing by hemostats. Hart et al. (2002) demonstrated that ORC/collagen modulates fibroblast activity in vitro and promotes granulation tissue formation and angiogenesis in vivo, suggesting its potential to support impaired wound healing [71]. In terms of gelatin-based dressings, according to an in vivo study by Schiefer et al. (2016), the repeated application of a gelatin-collagen non-woven dressing may serve as an effective treatment for chronic wounds, facilitating rapid wound closure through a combination of contraction and re-epithelialization [48]. These results align with the results from our animal study. All hemostats, but especially ORC and GELA, demonstrated increased proliferative, migration, and contractive capabilities. At the same time, ORC and GELA also demonstrated adequate dynamics for biomolecular mediators such as VEGF, TGF-β, and FGF-2. ONRC, in contrast, demonstrated opposing results to ORC and GELA, with potential negative implications for wound healing, such as a prolonged increased TGF-β level and reduced FGF-2 levels over time.

The observation that the applied hemostats did not negatively influence proliferation, α-SMA expression, VEGF, TGF-β, and FGF-2 levels within 14 days after application suggests that it may be considered safe for surgeons to leave the investigated hemostats in situ without jeopardizing the wound-healing process. Such could be the necessity in cases of significant bleeding, provided that postoperative wound healing is anticipated to proceed without complications. However, in the presence of risk factors for delayed wound healing, including obesity, revision surgery, or infection, caution may be warranted. Especially for ONRC, the potential later-stage negative effects on TGF-β and FGF-2 expression must be considered and could lead to excessive wound contraction or scarring. In this context, it would be useful to carry out comparative studies on wounds with a high risk of WHDs, investigating actual healing failure based on hemostat application.

These interpretations remain inferential, as gene expression was assessed in bulk tissue and cannot resolve cell-specific sources or functional consequences.

The present study was designed to address a clinically relevant postoperative question, namely whether absorbable hemostatic biomaterials, when left in situ after surgical wound closure, influence the early direction of wound remodeling. The selected time points of 14 and 30 days were chosen to capture a critical postoperative window in which wound healing either proceeds toward physiological maturation or begins to deviate toward pathological remodeling. Accordingly, the analyses focused on cellular and molecular markers indicative of remodeling direction rather than acute hemostatic effects or late-stage scar consolidation. While additional early or later time points may provide complementary information, the findings presented here specifically reflect early postoperative remodeling processes and were interpreted within this defined temporal scope.

## 5. Conclusions

Histological analyses demonstrated preserved tissue architecture across all groups, with no detectable remnants of cellulose-based materials at day 14. In contrast, the gelatin-based hemostat showed localized fibrous structures and visually increased extracellular matrix deposition, based on qualitative histological assessment.

All investigated hemostatic materials were well tolerated within the scope of this model during the early postoperative phase (up to day 14), with no apparent adverse effects on tissue morphology within the scope of this model. However, as no quantitative clinical outcome measures, complication assessment, or histomorphometric analyses were performed, conclusions regarding overall healing quality or clinical safety cannot be drawn.

At day 30, material-specific differences in cellular activity and growth factor expression were observed. GELA was associated with increased epidermal proliferation, while ORC and GELA showed higher α-SMA expression, and ONRC demonstrated elevated TGF-β expression. These findings suggest differences in cellular and molecular remodeling patterns between materials.

The present results should therefore be interpreted as indicative of differential tissue responses during early remodeling rather than definitive evidence of improved healing outcomes or long-term clinical effects. Further studies incorporating quantitative outcome measures, extended follow-up, and functional assessments are required to determine the clinical relevance of these observations.

## 6. Limitations

Several limitations of this study should be taken into account. First, Ki-67 serves as a non-specific marker of cell proliferation and does not enable identification of the specific proliferating cell populations; consequently, conclusions about the cellular sources of the observed proliferative response remain largely inferential. Second, the histological evaluation of residual biomaterial, ECM deposition, and fibrotic tissue was primarily descriptive, as neither a standardized semi-quantitative scoring system nor digital morphometric analyses were implemented. Third, gene expression analyses were performed on whole wound tissue, which may have obscured spatially restricted alterations at the biomaterial–tissue interface; additionally, early transient cytokine peaks may not have been detected at the chosen sampling intervals. Future studies should incorporate cell type-specific analyses, standardized quantitative histological assessments, and higher-resolution temporal and spatial profiling to better capture dynamic and localized biological responses. Furthermore, macroscopic wound-healing parameters were not quantitatively assessed. Although wound photographs were obtained at defined time points, no standardized evaluation of clinically relevant endpoints such as scar width, erythema, wound dehiscence, infection, or seroma/hematoma formation was performed. As a result, direct conclusions regarding clinically observable wound-healing quality remain limited. In addition, histological evaluation of tissue morphology and ECM deposition was primarily descriptive. No standardized histomorphometric or semi-quantitative scoring systems (e.g., collagen fraction, scar thickness, or fibrosis grading) were applied, which limits the ability to objectively quantify and compare structural differences between groups.

## Figures and Tables

**Table 1:** Investigated hemostatic biomaterials.

Agent	Abbr.	Product Name	Manufacturer
Oxidized non-regenerated cellulose	ONRC	*RESORBA* *^®^ CELL*	Resorba Medical GmbH, Nürnberg, Germany
Oxidized regenerated cellulose	ORC	*TABOTAMP^®^*	Ethicon, Johnson & Johnson Medical GmbH, Raritan, NJ, USA
Non-woven, porcine gelatine fabric	GELA	*GELITA TUFT-IT^®^*	GELITA medical GmbH, Eberbach, Germany

**Table 2:** Specification of termination criteria.

• extensive wound infection
• body weight reduction of ≥20%
• self-isolation
• pain or abnormal breathing sounds

## Data Availability

The datasets generated and/or analyzed during the current study are not publicly available because the raw data files contain sensitive experimental information not suitable for public sharing but are available from the corresponding author on reasonable request.

## Abbreviations

α-SMA	Alpha-smooth muscle actin
ECM	Extracellular matrix
EDTA	Ethylenediaminetetraacetic acid
ELISA	Enzyme-linked immunosorbent assay
FGF	Fibroblast growth factor
GELA	Gelatine-based hemostat (GELITA TUFT-IT^®^)
HE	Hematoxylin-eosin staining
ME	Mercaptoethanol
MP	Movat pentachrome staining
Ki-67	Kiel 67
PFA	Paraformaldehyde
ORC	Oxidized regenerated cellulose
ORNC	Oxidized non-regenerated cellulose
qPCR	Quantitative real-time polymerase chain reaction
TGFβ	Transforming growth factor β
VEGF	Tumor necrosis factor α Vascular endothelial growth factor
